# Risk stratification for endometrial cancer: independent and joint effects of polygenic risk score and body mass index in 129,829 UK Biobank participants

**DOI:** 10.1186/s12916-025-04570-5

**Published:** 2026-02-10

**Authors:** Xuemin Wang, Laure Dossus, Marc J. Gunter, Emma J. Davidson, Jue-Sheng Ong, Dylan M. Glubb, Tracy A. O’Mara

**Affiliations:** 1 Cancer Research Program, QIMR Berghofer, Brisbane, QLD 4059 Australia; 2 Nutrition and Metabolism Branch, International Agency for Research On Cancer (IARC/WHO), Lyon, 69007 France; 3 Cancer Epidemiology and Prevention Research Unit, School of Public Health, Imperial College London, London, UK; 4 Division of Cancer Sciences, Faculty of Biology, Medicine and Health, University of Manchester, St Mary’s Hospital, Manchester, M13 9WL UK; 5 Department of Obstetrics and Gynaecology, St Mary’s Hospital, Manchester Academic Health Science Centre, Manchester University NHS Foundation Trust, Manchester, M13 9WL UK; 6 Population Health Program, QIMR Berghofer, Brisbane, QLD 4059 Australia

**Keywords:** Endometrial cancer, Polygenic risk score, Body mass index, Risk stratification

## Abstract

**Background:**

Although obesity is a well-established risk factor for endometrial cancer, its relationship with genetic susceptibility in determining cancer risk remains unexplored. Current endometrial cancer risk prediction relies primarily on epidemiological factors, with limited consideration of genetic risk. We hypothesized that integrating polygenic risk score (PRS) information with established epidemiological factors could improve risk stratification and reveal whether genetic and lifestyle factors operate independently or jointly.

**Methods:**

We generated a polygenic risk score for endometrial cancer in 129,829 unrelated female participants of European genetic ancestry (including 956 incident cases with endometrial cancer) in the UK Biobank cohort. We evaluated the prediction model performance using area under the receiver operating characteristic curves (AUCs) and assessed individual and joint associations of body mass index (BMI) and PRS with endometrial cancer using Cox proportional hazards models.

**Results:**

The integrated model incorporating PRS and epidemiological risk factors achieved statistically significant improvement in predicting endometrial cancer compared with epidemiologic factors alone (AUC = 0.739 versus 0.728; *P* = 3.98 × 10^−5^). Participants in the top 1% PRS distribution had a 3.06-fold increased risk (95% CI 1.97–4.76), with a number needed to screen of 58 individuals. BMI and PRS demonstrated independent effects on endometrial cancer risk, with participants with a BMI ≥ 30 kg/m^2^ in the top PRS tertile showing the highest endometrial cancer risk (HR = 4.94; 95% CI 3.65–6.68). Even participants with a BMI < 25 kg/m^2^ in the top PRS tertile had a significantly increased risk (HR = 2.01; 95% CI 1.45–2.78).

**Conclusions:**

Integrating PRS with epidemiological risk factors provides potential for enhanced endometrial cancer risk stratification. PRS effects persist independently of BMI, suggesting genetic risk assessment could complement current screening approaches focused on Lynch Syndrome and identify additional high-risk individuals for targeted prevention strategies.

**Supplementary Information:**

The online version contains supplementary material available at 10.1186/s12916-025-04570-5.

## Background

Endometrial cancer is the most common gynecological cancer in developed countries, with 420,242 new cases and 97,704 new deaths estimated globally in 2022 [1]. Notably, over the last three decades, the incidence and mortality of endometrial cancer has been increasing worldwide [2].

Previous studies have used epidemiological risk factors, including age, BMI, parity, duration of oral contraceptive use, age at menarche, and age at menopause, to predict endometrial cancer status at moderate accuracy, with AUC values varying from 0.61 to 0.77 depending on the risk factors included and specific study populations [3–6]. Polygenic risk score (PRS) approaches, the cumulative dosage effects of multiple genetic variants identified by genome-wide association studies (GWAS), hold promise for disease stratification [7]. Incorporating polygenic risk scores into epidemiological models have achieved marginal improvement in the prediction of endometrial cancer development [3, 5, 6]. However, those studies have only included genome-wide significant or sub-genome-wide significant variants into the construction of the endometrial cancer PRS.

Obesity, typically measured by BMI, is the strongest known modifiable risk factor for endometrial cancer [8]. With the rising global prevalence of obesity [9], the incidence of endometrial cancer is further expected to increase. In addition to obesity and genetic variation, factors such as age, ages at menopause and menarche, parity and endogenous sex hormone levels also affect endometrial cancer risk [8, 10–19]. However, current endometrial cancer risk prediction relies primarily on epidemiological factors, with limited consideration of genetic risk. Integrating polygenic risk information with established epidemiological factors could substantially improve risk stratification and reveal whether genetic and lifestyle factors operate independently or jointly. Furthermore, identifying high-risk individuals beyond those screened for Lynch Syndrome could enable targeted prevention and screening strategies in clinical practice. This study addresses these gaps by as follows: (1) developing an integrated prediction model incorporating both PRS and epidemiological risk factors, (2) quantifying the independent and joint contributions of genetic risk and BMI to endometrial cancer development, and (3) evaluating whether genetic risk stratification could complement current clinical screening approaches in the UK Biobank cohort.

## Methods

### Study population

**Fig. 1:**
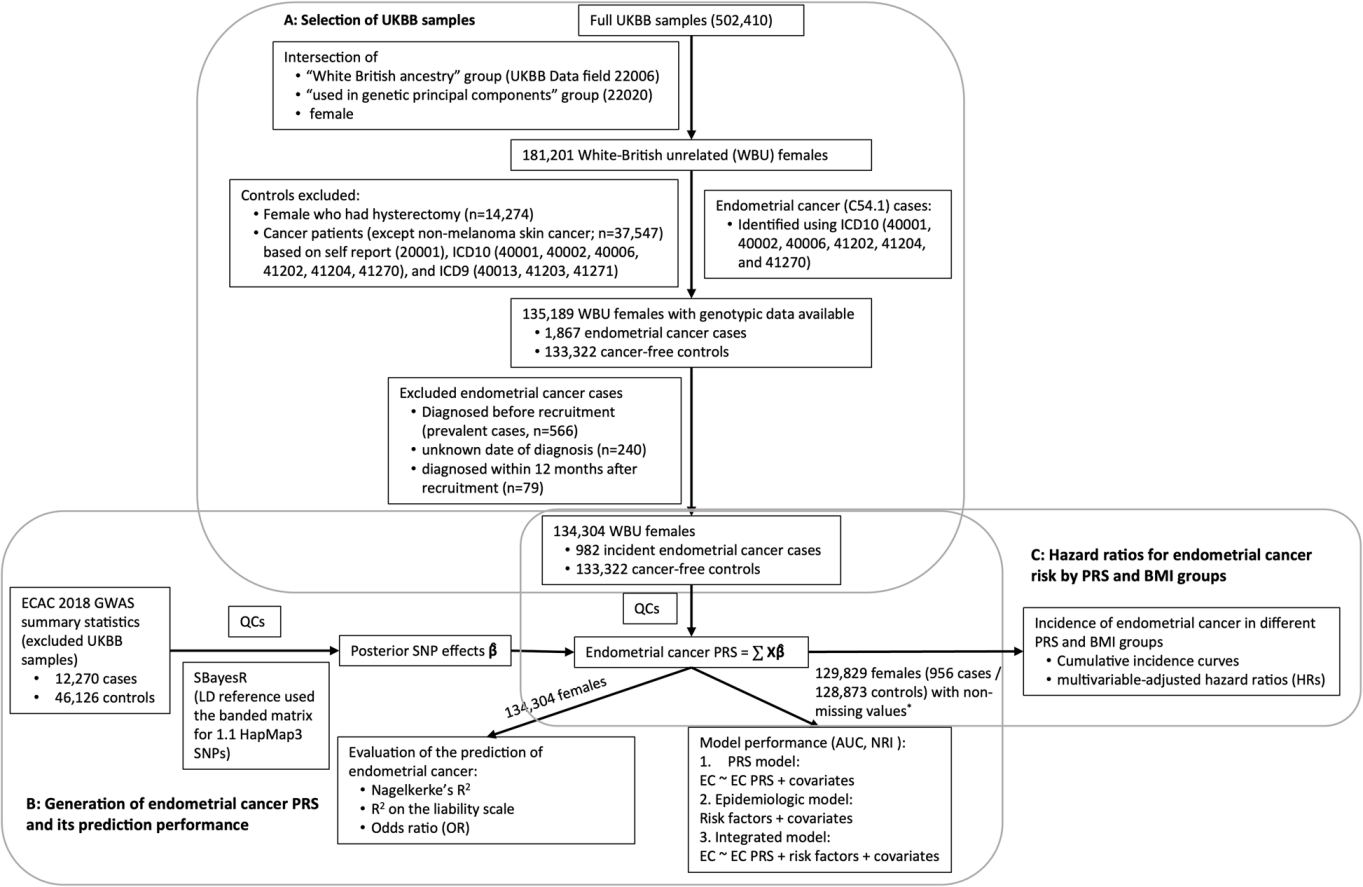
Flowchart for the selection of study participants in the UK Biobank and directed acyclic graph of the study design. (A) Flowchart of inclusion and exclusion criteria. (B) Flowchart of the generation of endometrial cancer polygenic risk scores (PRSs) and evaluation of its prediction of endometrial cancer status. (C) The directed acyclic graph for assessment of endometrial cancer incidence in different BMI and endometrial cancer PRS groups. QCs included filtered out SNPs with MAF < 0.01, multi-allelic variants, missing genotype rate in > 10% of samples, departing from Hardy–Weinberg Equilibrium (*P* < × 10^−6^), and low imputation quality (< 0.4). ^*^4475 females (26 cases and 4449 non-cases) within missing values of BMI, age at menarche, number of live births, or ever taken OC pill were excluded

A flowchart outlining the study design and population is presented in Fig. 1. This cohort study was based on data from the UK Biobank, which is a prospective cohort with extensive phenotypic and genotypic data for over 500,000 UK participants aged 40–70 at enrolment. Details of the UK Biobank can be found in Bycroft et al. [20, 21]. Briefly, participants were genotyped using either UK BiLEVE Axiom Array (807,411 genetic variants) or UK Biobank Axiom Array (825,927 genetic variants). Genetic variants were imputed using the 1000 Genomes phase 3, the UK10K, and the Haplotype Reference Consortium datasets as the imputation reference panels, which resulted in 93,095,623 autosomal single nucleotide polymorphisms (SNPs), short indels and large structural variants and 3,963,705 variants on the X chromosome.

### Selection of endometrial cancer cases and cancer-free participants

Following the exclusion of withdrawn participants, we restricted analyses to unrelated 181,201 female UK Biobank participants of European genetic ancestry, defined using both self-reported “White British” ancestry and genetic principal component clustering (UK Biobank Data Fields 22,006 and 22,020) created by Bycroft et al. [20] (Fig. 1A). While this approach reduces residual population structure and improves internal validity, it does not capture the full spectrum of human genetic diversity. In accordance with National Academies Science Engineering Medicine (NASEM) guidelines [22], we emphasize that the “White British” descriptor reflects a socially constructed category linked to the specific reference datasets used and should not be interpreted as a discrete or genetically homogenous population group. The use of this descriptor and principal component-based selection inherently limits the generalizability of our findings to other populations. Validation and, if necessary, recalibration of risk models in ancestrally and culturally diverse cohorts will be essential to ensure equitable and accurate risk prediction.

A total of 1867 endometrial cancer cases were identified among the 181,201 unrelated females of European genetic ancestry using the International Classification of Disease 10 (ICD10) subcategory C54.1 (malignant neoplasm of corpus uteri, Endometrium) in UK Biobank Data Fields 40,001 (underlying (primary) cause of death), 40,002 (contributory (secondary) causes of death), 40,006 (Type of cancer), 41,202 (diagnoses–main), 41,204 (diagnoses–secondary), and 41,270 (diagnoses). Diagnosis date was defined as the time of the first endometrial cancer record. From the unrelated females of European genetic ancestry, 133,322 females who had an intact uterus (no hysterectomy) and no prior cancer diagnosis (except non-melanoma skin) were included as controls. Prior cancers were identified based on ICD9 (40,013, 41,203, and 41,271); ICD10 (40,001, 40,002, 40,006, 41,202, 41,204 and 41,270); and self-reported cancers (20,001). We restricted analyses to 982 incident endometrial cancer cases. This included the removal of 566 prevalent cases who were diagnosed before recruitment, 240 cases without known date of diagnosis, and 79 cases who were diagnosed within 12 months to mitigate issues related to delayed diagnosis or delayed linkage to cancer registry.

To evaluate endometrial cancer risk prediction models (Fig. 1B, C), we additionally excluded 4475 females (26 cases and 4449 non-cases) with missing values of BMI, age at menarche, number of live births, or ever taken oral contraceptive (OC) pill, resulting in 956 cases and 128,873 cancer-free cohort participants.

### Generation of endometrial cancer PRS

GWAS summary statistics on endometrial cancer risk were sourced from the latest Endometrial Cancer Association Consortium (ECAC) GWAS analysis (12,906 cases and 108,979 controls), which included 636 endometrial cancer cases and 62,853 cancer-free female controls from the UK Biobank [13]. To avoid potential bias due to sample overlap between the GWAS dataset and the PRS validation dataset, the ECAC GWAS summary statistics were derived again by excluding UK Biobank samples, resulting in 12,270 endometrial cancer cases and 46,126 controls (Fig. 1B) [23].

All GWAS variants were directly genotyped or well imputed (imputation score > 0.4) and had a minor allele frequency (MAF) > 1%. Posterior effect sizes for genome-wide variants were derived using the SBayesR [24] method implemented in genome-wide complex trait Bayesian (GCTB) software [25]. The linkage disequilibrium (LD) matrix was computed based on 1.1 million HapMap 3 variants using a banded matrix with a window size of 3 cM per SNP in a random sample of 50,000 unrelated UK Biobank samples (https://cnsgenomics.com/software/gctb/#LDmatrices).

For UK Biobank individual-level genotypic data, standard GWAS quality controls were conducted to select genetic variants and samples for endometrial cancer PRS generation following the guidelines outlined by Choi et al. [26] (see https://choishingwan.github.io/PRS-Tutorial/). Briefly, genetic variants with a MAF < 0.01, a missing genotype rate exceeding 1%, or departing from Hardy–Weinberg Equilibrium (*P* < 1 × 10^−10^), were excluded, leaving 8,947,018 variants available for potential construction of the endometrial cancer risk PRS. All samples had no more than 10% missing genotypes. Per-individual PRS was calculated as the genome-wide sum of the per-variant posterior effect size multiplied by allele dosage using PLINK (https://www.cog-genomics.org/plink/) [27]. PRS values for individual females were centered to zero.

### Selection of established endometrial cancer risk factors

Drawing on insights from epidemiological and Mendelian randomization studies of endometrial cancer [11, 18, 28, 29], we selected and incorporated seven key risk factors into our prediction models for endometrial cancer status (case or non-case). The selected risk factors were as follows: measured BMI (UK Biobank data field 21,001), reported age at menarche (2714), age at natural menopause (3581), number of live births (2734), ever taken OC pill (2784), and levels of sex hormone binding globulin (SHBG; 30,830) and testosterone (30,850) during the initial assessment visit. The proportion of participants with missing information for variables was very low for BMI, age at menarche, number of live births, and ever taken OC pill (missingness range 0.3%–2.9%). The proportion of missing was greater for SHBG (14.3%) and testosterone levels (19.5%) likely due to issues associated with biospecimen processing, including sample quantity and quality failures. Age at menopause was missing for 3.4% of participants and not available for 42.6% of participants because they had not reached menopause.

In order to generate risk models that would be useful pre-menopausal, we generated a polygenic score (PGS) for age at menopause using the GWAS summary statistics provided by Ruth et al. (2021) [17, 30] and the same procedure as for the derivation for endometrial cancer PRS described above. We used age at menopause PGS in endometrial cancer risk prediction and in the analysis of associations of endometrial cancer with BMI and PRS. We similarly leveraged PGS methods for SHBG and testosterone levels. The use of PGS for these factors in risk prediction models will allow for replication in other cohorts that typically do not have these measured. PGS for levels of SHBG and testosterone were generated using the female-stratified GWAS summary statistics provided by Ruth et al. [29, 31, 32] and the same procedure as for the derivation for endometrial cancer PRS. The resulting PGS values for SHBG and testosterone instead of their measured values were included as covariates to assess the performance of our prediction models and in the analysis of associations of endometrial cancer with BMI and PRS. To address potential circularity or bias from including PGS derived from partially overlapping datasets, we performed a sensitivity analysis repeating the main analysis using measured SHBG and testosterone levels and reported age at menopause in place of their corresponding PGS. These sensitivity models were constructed within the same UK Biobank cohort (557 incidence cases and 50,303 controls with all measurements available) to evaluate whether the inclusion of genetically proxied traits materially affected associations or prediction performance.

Age at initial assessment (UK Biobank data field 21,003) and the top 10 genetic principal components (PCs) were included in all prediction models as covariates. To estimate genetic PCs, a genetic relationship matrix among individuals was created using HapMap3 variants and the GCTA-GREML method [33]. The genetic relationship matrix was used to derive genetic PCs using the GCTA software (version 1.94.1) [34].

### Evaluation of PRS model performance and established risk factors

We assessed the predictive performance of the endometrial cancer PRS model in the UK Biobank using logistic regression to calculate the Nagelkerke’s *R*^2^ and variance on the liability scale explained by PRS as described previously [35]. We converted the observed *R*^2^ to the liability scale assuming an endometrial cancer population prevalence of 3%. Participants were divided into percentiles based on their endometrial cancer PRS distribution, and their estimated odds ratios (ORs) for endometrial cancer risk were calculated using the middle two deciles (40%–60%) as the reference group. AUCs were reported for the endometrial cancer PRS and the following: (1) each of the seven established endometrial cancer risk factors; (2) the epidemiologic model comprising all seven established endometrial cancer risk factors; and (3) an integrated model combining the epidemiological model with the endometrial cancer PRS. All analyses were adjusted for age at initial visit and the top 10 genetic PCs. We used stratified bootstrap with 2000 replicates to compute their corresponding 95% confidence intervals (CIs). Sample size adequacy was assessed using the events-per-variable (EPV) approach [36]. With 982 endometrial cancer cases and 8 predictors in the integrated model (7 epidemiological variables plus PRS), our study achieved an EPV of 122.8, exceeding the minimum threshold of 10 events per variable recommended by Peduzzi et al. [36]. Number needed to screen (NNS) was calculated as the reciprocal of absolute risk for each PRS percentile to provide clinically interpretable risk stratification metrics [37]. We calculated the net reclassification index (NRI) to quantify the improvement in the reclassification of endometrial cancer cases and non-cases by the integrated model as compared to the traditional epidemiological model (i.e. without PRS). NRI was interpreted as the net proportion of participants with improved risk classification, following established guidance for clinical prediction model evaluation [38].

### BMI and PRS associations with endometrial cancer

UK Biobank incident endometrial cancer cases and non-cases were categorized by BMI (BMI < 25 kg/m^2^, 25 kg/m^2^ ≤ BMI < 30 kg/m^2^, and BMI ≥ 30 kg/m^2^) or endometrial cancer PRS tertiles from non-cases. We evaluated associations of BMI and endometrial cancer PRS with endometrial cancer status using Cox proportional hazard models. We tested the proportional hazards assumption for covariates included in a model fit by testing for independence between the scaled Schoenfeld residuals and time. *P*-values for trend were estimated using endometrial cancer PRS and BMI as continuous variables. The multivariable models were adjusted with each other for BMI groups and endometrial cancer PRS tertiles and additionally accounted for age at menarche, number of live births (as a categorical factor), ever taken oral contraceptive pill, PGS for age at menopause, PGS for SHBG levels, PGS for testosterone levels, age at initial assessment, and the top 10 genetic PCs. We additionally performed a sensitivity analysis where multivariable models were adjusted with each other for BMI and endometrial cancer PRS as continuous variables. Follow-up time was calculated from the baseline date (date when attended assessment center during their initial visit) to the date of endometrial cancer diagnosis or death (whichever occurred first). Cumulative incidence rates of endometrial cancer during the follow up period were generated for the different BMI and endometrial cancer PRS groups. We tested the interactions between BMI and endometrial cancer PRS by adding an interaction term in the multivariable model.

We further explored the joint associations of BMI groups and PRS tertiles with endometrial cancer using the Cox proportional model accounting for all covariates mentioned above. A nine-group comprehensive variable was thus formed, and the first PRS tertile and a normal weight (BMI < 25 kg/m^2^) group was considered as the reference group. We used a Z-test comparison to assess for differences in PRS effect between groups, as per Reay et al. [39].

All analyses were performed using R software version 4.2.2 (https://www.R-project.org/). Distribution of baseline characteristics was assessed by descriptive statistics and compared between case and non-case groups using *χ*^2^ tests for categorical variables and *t*-tests for continuous variables. Cox proportional hazards ratio models were conducted using the survival (version 3.5–5) and cumulative incidence rate plots were generated using the survminer (version 0.4.9) R packages. NRI calculations were performed using the PredictABEL R package (version 1.2–4) [40]. AUC were assessed using the pROC (version 1.18.0) R package [41]. Statistical tests were two-sided and statistical significance was evaluated at *P* < 0.002 after Bonferroni correction for 25 post hoc comparisons. The correction encompasses the primary endometrial cancer PRS association, PRS decile contrasts, model AUC comparison, BMI- and PRS-stratified risk analyses, and joint BMI × PRS cell contrasts. *P*-values for baseline characteristic were considered descriptive. Data analysis was conducted from February 22 to Sept 21, 2023.

## Results

### Baseline characteristics of study population

This analysis comprised 134,304 unrelated female participants of European genetic ancestry, including 982 cases of endometrial cancer and 133,322 cancer-free cohort participants (Fig. 1A). Consistent with established knowledge, endometrial cancer cases, in comparison to non-cases, exhibited older age at initial assessment, earlier age at menarche, later age at menopause, lower SHBG levels, higher testosterone levels, and a lower frequency of oral contraceptive pill use (Table 1; Additional File 1: Table S1). While the mean number of live births did not significantly differ between cases and controls, a higher proportion of cases were nulliparous compared to non-cases (22.6% vs. 18.9%). As anticipated, we observed a higher prevalence of obesity (BMI ≥ 30 kg/m^2^) among participants with endometrial cancer compared to non-cases (*P* < 2.2 × 10^−16^; Table 1). Although several baseline variables reached statistical significance, many of these reflect small absolute differences that are not clinically meaningful and are expected given the large cohort size. While differences such as BMI and oral contraceptive pill use reflect large, clinically relevant associations, variables with smaller differences were nevertheless included as covariates to ensure proper control for confounding in subsequent multivariable analyses.

**Table 1:** Baseline characteristics of participants in the UK Biobank

Baseline characteristics	Overall	Cases	Controls	*P*-value
Number of female participants	13,4304	982 (0.7%)	133,322 (99.3%)	
Age at initial assessment, years (SD)	55.7 (8.0)	59.5 (6.6)	55.7 (8.0)	< 2.2 × 10^−16^
BMI (SD), continuous	26.9 (5.1)	30.5 (6.9)	26.9 (5.1)	< 2.2 × 10^−16^
BMI categories^a^				
BMI < 25 kg/m^2^	55,090 (41.0%)	210 (21.4%)	54,880 (41.2%)	< 2.2 × 10^−16^
25 kg/m^2^ < = BMI < 30 kg/m^2^	48,703 (36.3%)	340 (34.6%)	48,363 (36.3%)	
BMI > = 30 kg/m^2^	30,128 (22.4%)	427 (43.5%)	29,701 (22.3%)	
Missing (%)	383 (0.3%)	5 (0.5%)	378 (0.3%)	
Age at menarche, years (SD)	13.0 (1.6)	12.7 (1.6)	13.0 (1.6)	< 9.5 × 10^–10^
Number of females who reported their age at menarche (%)	130,449 (97.1%)	961 (97.9%)	129,488 (97.1%)	
Missing (%)	3855 (2.9%)	21 (2.1%)	3834 (2.9%)	
Age at menopause, years (SD)	50.4 (4.4)	51.7 (4.3)	50.4 (4.4)	< 2.2 × 10^−16^
Number of post-menopausal females (%)	72,459 (54.0%)	754 (76.8%)	71,705 (53.8%)	
Number of pre-menopausal females (%)	57,218 (42.6%)	184 (18.7%)	57,034 (42.8%)	
Missing (%)	4627 (3.4%)	44 (4.5%)	4583 (3.4%)	
Age at menopause PGS^b^ (SD)	0 (1.43)	0.17 (1.44)	0 (1.43)	2.1 × 10^−4^
SHBG levels (nmol/L; SD)	62.5 (30.7)	50.6 (26.0)	62.6 (30.7)	< 2.2 × 10^−16^
Number of females that had detectable SHBG levels	115,035 (85.7%)	854 (87.0%)	114,181 (85.6%)	
Missing (%)	19,269 (14.3%)	128 (13.0%)	19,141 (14.4%)	
SHBG PGS^b^ (SD)	0 (0.22)	−0.05 (0.23)	0 (0.22)	8.2 × 10^−11^
Testosterone levels (nmol/L; SD)	1.1 (0.6)	1.2 (0.6)	1.1 (0.6)	5.4 × 10^−7^
Number of females that had detectable testosterone levels	108,095 (80.5%)	820 (83.5%)	107,275 (80.5%)	
Missing (%)	26,209 (19.5%)	162 (16.5%)	26,047 (19.5%)	
Testosterone PGS^b^ (SD)	0 (0.34)	0.05 (0.36)	0 (0.34)	2.8 × 10^−6^
Number of live births (SD), count	1.8 (1.2)	1.7 (1.2)	1.8 (1.2)	0.2
Number of live births categories^a^				
0 (%)	25,428 (18.9%)	222 (22.6%)	25,206 (18.9%)	1.1 × 10^−2^
1 (%)	17,798 (13.3%)	118 (12.0%)	17,680 (13.3%)	
> 1 (%)	90,999 (67.8%)	642 (65.4%)	90,357 (67.8%)	
Missing (%)	79 (0.1%)	0	79 (0.1%)	
Ever taken oral contraceptive pill^a^				
Yes (%)	112,428 (83.7%)	712 (72.5%)	111,716 (83.8%)	< 2.2 × 10^−1^
No (%)	21,645 (16.1%)	270 (27.5%)	21,375 (16.0%)	
Missing (%)	231 (0.2%)	0	231 (0.2%)	

*Abbreviations*: *SD* Standard deviation, *BMI* Body mass index, *PGS* Polygenic score, *SHBG* Sex hormone binding globulin

^a^Percentages may not add up to 100% due to rounding

^b^PGS (polygenic scores) of age at menopause, SHBG, and testosterone were shifted to mean zero

### Integration of PRS and established risk factors for the prediction of endometrial cancer

The endometrial cancer PRS was strongly associated with endometrial cancer risk amongst unrelated participants with European genetic ancestry (*P*-value < 2.2 × 10^−16^) (Additional File 2: Figure S1). The Nagelkerke’s *R*^2^, a pseudo-*R*^2^ statistic measuring proportion of variance explained by endometrial cancer PRS, was 0.9% and variance on the liability-scale explained by endometrial cancer PRS was 1.9%. Risk stratification analysis revealed meaningful differences across PRS percentiles. Participants in the top 1% of the distribution had an absolute endometrial cancer risk of 1.71%, corresponding to an NNS of 58. The top 10% showed 1.05% absolute risk with NNS of 95. Compared to the middle quintile of participants (40–60%), participants in the top 10% and top 1% of the PRS distribution had a 1.98-fold (95% CI 1.58–2.49; *P* = 3.03 × 10^−9^) and 3.06-fold (95% CI 1.97–4.76; *P* = 7.10 × 10^−7^) increased risk of developing endometrial cancer respectively (Table 2). The PRS model predicted endometrial cancer status at an AUC of 0.671 (95% CI 0.654–0.687). While this was slightly higher than for six of the established endometrial cancer risk factors (AUC range 0.648–0.659), it was lower than the prediction accuracy for BMI (AUC = 0.713; 95% CI 0.696–0.729) (Table 3). The integrated model achieved superior discrimination compared to the epidemiological model alone (AUC = 0.739 vs 0.728; difference = 0.011; *P* = 3.98 × 10^−5^), representing a 1.5% relative improvement. The continuous net reclassification improvement for endometrial cancer prediction was 0.25 (95% CI 0.19–0.31; *P* < 1 × 10^−4^), indicating that for every 1000 participants screened, the integrated model would provide net improved risk classification for 250 participants compared with the epidemiological model alone.

**Table 2:** Risk of endometrial cancer across polygenic risk score (PRS) percentiles, including absolute risk and number needed to screen (NNS)

**Percentile**	**OR**	**95% CI**	**P-value**	**Cases (n)**	**Non-cases (n)**	**Absolute risk (%)**	**NNS**
0–10%	0.64	0.47 - 0.89	0.007	49	13,382	0.37	274
10–20%	1.04	0.79 - 1.37	0.78	79	13,351	0.59	170
20–30%	1.08	0.82 - 1.41	0.58	82	13,348	0.61	164
30–40%	1.16	0.89 - 1.51	0.27	88	13,343	0.66	153
40–60% (Reference)	1	NA	NA	152	26,708	0.57	177
60–70%	1.60	1.26 - 2.03	1.3 × 10^-^^4^	121	13,310	0.90	111
70–80%	1.65	1.30 - 2.09	3.5 × 10^−5^	125	13,305	0.93	107
80–90%	1.80	1.42 - 2.27	7.5 × 10^−7^	136	13,294	1.01	99
90–100%	1.98	1.58 - 2.49	3.0 × 10^−9^	127	11,960	1.05	95
99–100%	3.06	1.97 - 4.76	7.1 × 10^−7^	23	1,321	1.71	58

Endometrial cancer risk by decile of polygenic risk score (PRS) among 129,829 unrelated UK Biobank women of European genetic ancestry, with the 40–60th percentile used as the reference category. The number needed to screen (NNS) was calculated as the reciprocal of absolute risk, estimating how many women would need to be screened to detect one case of endometrial cancer

*Abbreviations*: *OR* Odds ratio, *CI* Confidence interval, *NNS* Number needed to screen

**Table 3:** Comparison of endometrial cancer prediction model performance using area under the curve (AUC), sensitivity, and specificity

**Prediction Model**	**AUC**	**95% CI**	**Sensitivity (%)**	**Specificity (%)**	**PPV (%)**	**NPV (%)**
Endometrial cancer PRS	0.671	0.654–0.687	66.0	58.8	1.2	99.6
BMI (continuous)	0.713	0.696–0.729	63.9	67.6	1.4	99.6
Number of live births	0.648	0.632–0.664	60.5	60.1	1.1	99.5
Age of menarche	0.655	0.639–0.670	61.8	60.7	1.2	99.5
Age of menopause PGS	0.648	0.632–0.664	62.9	58.9	1.1	99.5
OCP use (never/ever)	0.652	0.637–0.668	62.8	58.9	1.1	99.5
SHBG level PGS	0.659	0.644–0.675	65.0	58.2	1.1	99.6
Testosterone level PGS	0.652	0.636–0.668	61.8	60.1	1.1	99.5
Epidemiological model	0.728	0.712–0.744	68.3	64.7	1.4	99.6
Integrative model	0.739	0.723–0.754	63.3	71.6	1.6	99.6

Discriminative performance of endometrial cancer risk factors and prediction models in UK Biobank. Epidemiological model incorporated established risk factors (BMI, age at menarche, parity, oral contraceptive use, menopause age, SHBG, and testosterone); integrative model combining PRS with the epidemiological model. All models were adjusted for age at initial visit, assessment centre and the first ten principal components

*Abbreviations*: *AUC* Area under the receiver operator curve, *CI* Confidence interval, *PPV* Positive predictive value, *NPV* Negative predictive value, *PRS* Polygenic risk score, *BMI* Body mass index, *PGS* Polygenic score, *OCP* Oral contraceptive pill, *SHBG* Sex hormone binding globulin

### Associations of BMI and PRS with endometrial cancer risk

**Fig. 2:**
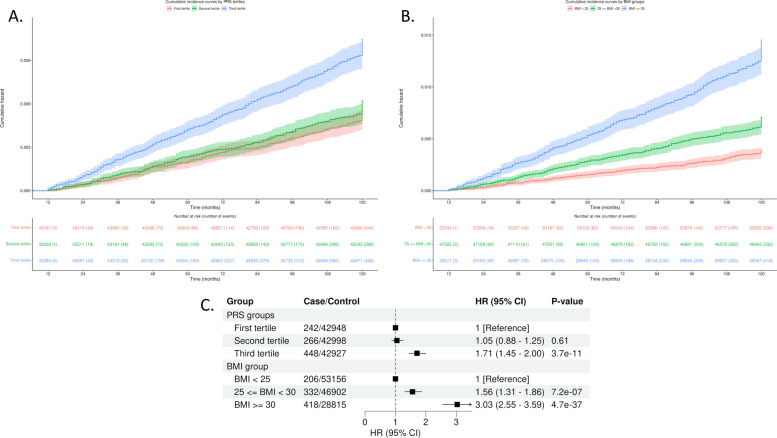
Cumulative incidence curves and multivariable-adjusted effects of endometrial cancer polygenic risk score (PRS) and BMI on endometrial cancer risk. Cumulative incidence curves were drawn for PRS tertiles (**A**) and BMI groups (**B**) in the UK Biobank. The unit of follow-up time was months, and the start point was defined as the 12th month (1 year after recruitment). **C** Multivariable models were adjusted for either PRS or BMI group and additionally adjusted for age at menarche, number of live births, ever taken oral contraceptive pill, and PGS values of age at menopause and levels of SHBG and testosterone, as well as age at initial assessment and the top 10 genetic principal components

The proportional hazards regression model assumes that the ratio of hazards between two groups is constant over time. There was no evidence to support violation of the proportional hazard assumption in the current analysis (global test *P* = 0.08). Cumulative incidence curves indicated that only participants in the top tertile of the PRS distribution displayed increased risk compared to the bottom and middle tertiles; whereas higher cumulative incidence rate was associated with both the overweight (25 kg/m^2^ ≤ BMI < 30 kg/m^2^) and obese (BMI ≥ 30 kg/m^2^) groups (Fig. 2A). BMI and PRS were independently associated with endometrial cancer risk (Fig. 2B). In the model mutually adjusted for BMI groups and PRS tertiles, compared with the bottom PRS tertile, the top PRS tertile displayed an increased risk (1.71-fold) for endometrial cancer (95% CI, 1.45–2.00; *P* = 3.7 × 10^−11^). For BMI, overweight and obese groups presented a 1.56-fold (95% CI, 1.31–1.86; *P* = 7.2 × 10^−7^) and a 3.03-fold (95% CI, 2.55–3.59; *P* = 4.7 × 10^−37^), increased risk of endometrial cancer, respectively, compared with normal BMI group (Fig. 2B).

**Fig. 3:**
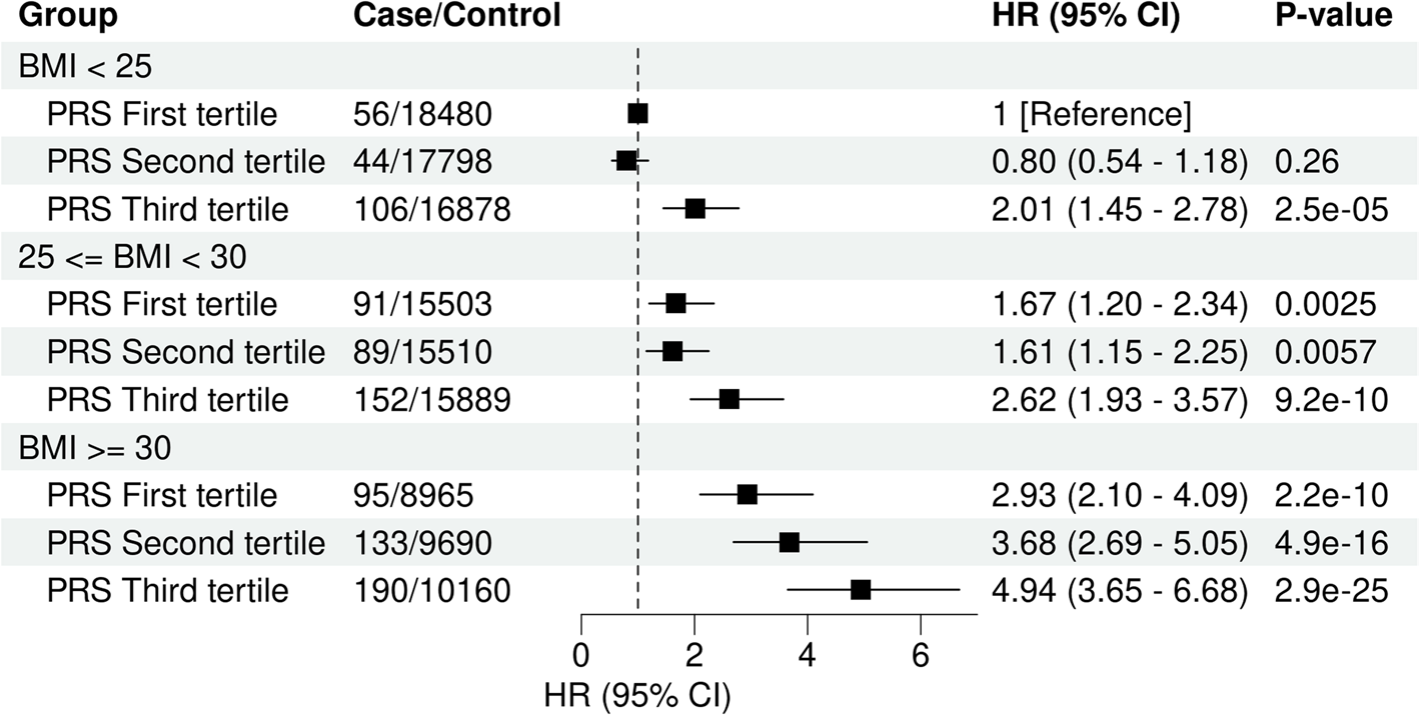
The joint association of genetic risk and BMI with endometrial cancer. Multivariable models were adjusted for age at initial assessment, age at menarche, number of live births, ever taken oral contraceptive pill, PRS values of age at menopause and SHBG and testosterone and the top 10 genetic principal components. The dashed vertical line indicates a Hazard ratio of 1

When endometrial cancer PRS and BMI were treated as continuous variables, an increase of one standard deviation of endometrial cancer PRS was associated with 2.13-fold risk of endometrial cancer (95% CI, 1.79–2.54; *P* = 2.52 × 10^−17^), while a one standard deviation increase of BMI (5.13 kg/m^2^) was associated with a 1.59-fold risk (95% CI, 1.52–1.67; *P* = 2.52 × 10^−80^). There was no evidence of interaction between BMI and endometrial cancer PRS (*P* for interaction = 0.39). Upon stratification by BMI group, we observed that only those with the greatest polygenic load (i.e. the top PRS tertile) in each group had an increased risk of endometrial cancer risk. Participants in the top PRS tertile and the highest BMI group exhibited the greatest risk (HR = 4.94; 95% CI, 3.65–6.68; *P* = 7.67 × 10^−25^; Fig. 3); the relatively wide confidence intervals likely reflecting fewer incident cases in this subgroup and additional variability from stratifying on two correlated risk factors. Even for participants with a normal BMI, those in the top PRS tertile had a 2.01-fold increased risk (95% CI, 1.45–2.78; *P* = 2.50 × 10^−5^), compared with the bottom PRS tertile. Supporting the independent effects of BMI and PRS on endometrial cancer risk, the effect of the PRS in the highest BMI group was not significantly different to the PRS effect in the lowest BMI group (Z-score for difference in effect sizes = 1.59; *P*-value = 0.11). Increased risks for the top PRS tertile persisted after additionally adjusting for continuous BMI (Additional File 2: Figure S2).

In sensitivity analyses, replacing PGS for SHBG, testosterone, and age at menopause with their directly measured phenotypes, the results were highly consistent with our main findings. The integrated model combining the endometrial cancer PRS with measured epidemiological factors achieved an AUC of 0.753 (95% CI 0.733–0.773) compared with an AUC of 0.736 (CI 95% 0.715–0.757) for the epidemiological model alone (*P* = 7.9 × 10^−5^), mirroring the results using PGS (AUC = 0.739 vs 0.728; Table 3). Joint BMI × PRS assessment was also unchanged. The full results are provided in Additional File 3: Supplementary Note.

## Discussion

In this cohort study of the UK Biobank, our initial focus was on assessing the predictive performance of endometrial cancer PRS and established risk factors. Firstly, we generated a risk distribution for PRS, revealing that individuals in the top 1–10% of the PRS had a risk comparable to that associated with a first-degree family history of endometrial cancer [42, 43]. The incorporation of PRS with the epidemiological risk factors in a risk prediction model led to a modest improvement in performance compared to the model that included the non-genetic risk factors alone. Subsequently, we evaluated associations of BMI and PRS with endometrial cancer, finding a joint association with endometrial cancer risk, with participants with high BMI who also had the highest PRS exhibiting the greatest risk. Participants in the top PRS tertile experienced a lower risk if they were not obese. Conversely, individuals in the top PRS tertile had increased endometrial cancer risk compared to other tertiles, even when they had a normal weight. These findings highlight the potential use of endometrial cancer PRS to identify high-risk individuals in the UK Biobank, which is particularly significant given the lack of clinical guidelines for endometrial cancer screening in the general population [44]. Indeed, current screening recommendations are primarily targeted at women with or at risk of Lynch syndrome who account for only ~ 3% of endometrial cancer cases [45, 46].

Previous studies have shown that PRS alone provides limited benefit in population screening, individual risk prediction, and population risk stratification [47, 48]. However, disease risk stratification can be improved through the integration of PRS and other risk factors [49], as we have also demonstrated. One more appropriate application of PRS is to facilitate personalized cancer screening and management practices. For instance, inclusion of PRS into breast cancer risk estimation may reduce screening and enable more personalized risk management strategies for *CHEK2* and *ATM* pathogenic variant carriers [50]. PRS has also been shown to add value in distinguishing type 1 from type 2 and monogenic diabetes in adults with a pre-existing diagnosis of diabetes and in selecting the best treatment for different types of diabetes [51]. As we observed marginal improvement by incorporating endometrial cancer PRS with established risk factors, endometrial cancer PRS may aid in stratifying individuals with Lynch syndrome pathogenic variants.

To the best of our knowledge, this is the first study to quantify the association of BMI with endometrial cancer risk across different genetic risk levels defined by PRS. Our results suggest that, irrespective of PRS, endometrial cancer risk is positively associated with BMI and can be substantially mitigated by weight reduction. An analysis of the Women’s Health Initiative observational study found that women experiencing weight loss of ≥ 5% was linked to a 29% decrease in endometrial cancer risk, and intentional weight loss of ≥ 5% in females with obesity was associated with 56% lower endometrial cancer risk [52]. A separate meta-analysis of 13 published studies confirmed a similar association between intentional weight loss and reduced endometrial cancer risk; additionally, the analysis revealed that bariatric surgery was associated with a remarkable 59% reduction in endometrial cancer risk [53].

The findings that endometrial cancer risk associates with a higher PRS after accounting for established risk factors suggest that endometrial cancer can progress via other mechanisms. Furthermore, although obesity is often defined by BMI, it does not fully represent the distribution of body fat within the human body. In a recent study, it was reported that BMI formed a distinct cluster of fat distribution traits, such as visceral-to-subcutaneous adipose tissue ratio, waist-to-hip ratio without adjustment for BMI, and waist adjusted for BMI. The study further revealed that one unit increase in waist-to-hip ratio or visceral-to-subcutaneous adipose tissue ratio was associated with elevated hazard ratio for endometrial cancer [54]. Therefore, including other aspects of obesity such as fat distribution and other risk factors not assessed in this study such as duration of hormonal therapy usage, LDL-cholesterol levels, circulating estrogen levels, sedentary behaviors, and smoking (summarized in [10, 11]) may further improve our capability to predict endometrial cancer in the general population.

The observed risk stratification has practical implications for endometrial cancer prevention. The 3.06-fold increase in the top 1% PRS represents a substantial genetic risk factor affecting 1% of the population, compared to mismatch repair pathogenic variants associated with Lynch Syndrome, which affects approximately 0.3% of the general population [55] yet account for only ~ 3% of endometrial cancer [42, 43]. This genetic risk stratification could identify high-risk individuals beyond the current focus on Lynch Syndrome screening. The NNS of 58 for the highest-risk PRS group compares favorably to established fecal occult blood test screening programs for colorectal cancer, where Australian data suggest an NNS of approximately 33 to detect a colorectal cancer case via colonoscopy among participants testing positive for microscopic blood in the stool [56].

We acknowledge that using PGS derived from partially overlapping datasets may introduce bias through circularity or collider mechanisms. However, our sensitivity analyses using directly measured hormone levels and reported age at menopause produced results consistent with those obtained using PGS, supporting that any potential overlap did not materially influence the associations observed.

Our study has several limitations. First, our findings lack external validation in an independent cohort, limiting the assessment of model generalizability outside the UK Biobank population. While the large sample size and robust EPV of 122.8 provides confidence in model stability, future work should validate both the PRS and the integrated model in external cohorts. Second, while the statistically significant improvement in the integrated model AUC is modest, the clinical impact of this enhancement is supported by a continuous NRI of 0.25, equivalent to ~ 250 participants correctly reclassified per 1000 screened, underscoring the value of adding genetic risk information to traditional epidemiological models. Future studies, such as decision curve and cost-effectiveness analyses, could further evaluate the clinical utility of this integrated model. Third, our analysis was restricted to participants of European genetic ancestry, defined using UK Biobank’s combined self-reported and genetic criteria, to minimize confounding from population structure. However, as emphasized in the NASEM report [22], these population descriptors do not reflect discrete or genetically homogenous groups, but are shaped by sampling frameworks and sociocultural context. This approach enhances internal validity but restricts generalizability; predictive performance may attenuate, and risk associations may differ in non-European or admixed populations due to underlying differences in allele frequencies, linkage disequilibrium, and genetic architecture. We fully acknowledge that principal component analysis and self-reported ancestry are imperfect proxies for human genetic diversity and may mask important variation, as outlined in current guidelines. Validation and recalibration of polygenic risk scores in ancestrally and culturally diverse cohorts are essential to ensure equitable and clinically useful risk prediction. Fourth, BMI was assessed at a single timepoint, which may misclassify exposure and attenuate associations if weight changed during follow-up; future analyses using repeated BMI assessments in UK Biobank with time-updated Cox models, longitudinal weight-change trajectories, or joint models of longitudinal BMI and incident endometrial cancer could better capture dynamic adiposity and refine risk estimates.

## Conclusions

The findings of this study demonstrate that an endometrial cancer prediction model incorporating both epidemiological risk factors and PRS has the best performance. The improvement of prediction of endometrial cancer by PRS is anticipated to increase as additional genetic risk variants are identified by larger GWAS. Importantly, BMI and endometrial cancer PRS exert independent effects on the risk of developing endometrial cancer, revealing a substantial increase in risk for individuals with highest BMI and high PRS. While underscoring the potential protective impact of weight loss, our findings also indicate that elevated PRS poses an increased risk, even in individuals with a normal weight. These insights emphasize the complex interplay between genetic susceptibility, lifestyle factors, and obesity in endometrial cancer risk, reinforcing the need for personalized and nuanced approaches to screening and preventive interventions.

## Supplementary Information

Additional file 1: Table S1. Baseline characteristics of the 129,829 UK Biobank female participants included in the study, stratified by endometrial cancer case status. Characteristics include demographic data, anthropometric measurements, reproductive factors, and hormone levelsand their corresponding polygenic scores.

Additional file 2: Supplementary Figures. Fig S1–Distribution of the endometrial cancer polygenic risk scoreby unrelated female cases and controls of European genetic ancestry in UK Biobank. Fig S2–Joint association of genetic risk and BMI with endometrial cancer risk with additional adjustment for continuous BMI.

Additional file 3: Supplementary Note. Sensitivity analyses using reported age of menopause and measured SHBG and testosterone levels instead of polygenic scores for these variables. Includes Table 1 comparing discriminative performance of prediction models and Figs. 1–2 demonstrating that BMI and PRS independently associate with endometrial cancer risk without evidence of interaction.

Additional file 4: Supplementary Note. Strengthening the Reporting of Observational Studies in Epidemiology checklist.

## Data Availability

Access to the UK Biobank data used this in study is available through application to the UK Biobank in accordance with their established access procedure (https://www.ukbiobank.ac.uk/enable-your-research/apply-for-access). Endometrial cancer GWAS summary statistics used to generate endometrial cancer polygenic risk scores can be downloaded from the GWAS Catalog (Study accession: GCST006464) [57, 58]. GWAS summary statistics used to generate polygenic scores for age at natural menopause can be downloaded from the GWAS Catalog (Study accession: GCST90320256) [30]. Female-stratified GWAS summary statistics used to generate polygenic scores for SHBG and testosterone can be downloaded from the GWAS Catalog (Study accession: GCST90012107 for SHBG and GCST90012112 for testosterone) [31, 32].

## Abbreviations

AUC: Area under the receiver operator curve
BMI: Body mass index
CI: Confidence interval
ECAC: Endometrial Cancer Association Consortium
EPV: Events per variable
GCTB: Genome-wide complex trait Bayesian
GWAS: Genome-wide association study
ICD10: International classification of disease 10
LD: Linkage disequilibrium
MAF: Minor allele frequency
NNS: Number needed to screen
NRI: Net reclassification index
OC: Oral contraceptive
PC: Principal components
PGS: Polygenic score
PRS: Polygenic risk score
SHBG: Sex hormone binding globulin
SNP: Single nucleotide polymorphism

## References

[1] Bray F, Laversanne M, Sung H, Ferlay J, Siegel RL, Soerjomataram I, et al. Global cancer statistics 2022: GLOBOCAN estimates of incidence and mortality worldwide for 36 cancers in 185 countries. CA Cancer J Clin. 2024;74(3):229–63. 38572751 10.3322/caac.21834

[2] Gu B, Shang X, Yan M, Li X, Wang W, Wang Q, et al. Variations in incidence and mortality rates of endometrial cancer at the global, regional, and national levels, 1990-2019. Gynecol Oncol. 2021;161(2):573–80. 33551200 10.1016/j.ygyno.2021.01.036

[3] Bafligil C, Thompson DJ, Lophatananon A, Ryan NAJ, Smith MJ, Dennis J, et al. Development and evaluation of polygenic risk scores for prediction of endometrial cancer risk in European women. Genet Med. 2022;24(9):1847–56. 35704044 10.1016/j.gim.2022.05.014

[4] Hüsing A, Dossus L, Ferrari P, Tjonneland A, Hansen L, Fagherazzi G, et al. An epidemiological model for prediction of endometrial cancer risk in Europe. Eur J Epidemiol. 2016;31(1):51–60. 25968175 10.1007/s10654-015-0030-9

[5] Kachuri L, Graff RE, Smith-Byrne K, Meyers TJ, Rashkin SR, Ziv E, et al. Pan-cancer analysis demonstrates that integrating polygenic risk scores with modifiable risk factors improves risk prediction. Nat Commun. 2020;11(1):6084. 33247094 10.1038/s41467-020-19600-4 PMC7695829

[6] Shi J, Kraft P, Rosner B, Benavente Y, Black A, Brinton LA, et al. Risk prediction models for endometrial cancer: development and validation in an international consortium. J Natl Cancer Inst. 2023;115(5):552–9. 10.1093/jnci/djad014 PMC10165481 36688725

[7] Torkamani A, Wineinger NE, Topol EJ. The personal and clinical utility of polygenic risk scores. Nat Rev Genet. 2018;19(9):581–90. 29789686 10.1038/s41576-018-0018-x

[8] Setiawan VW, Yang HP, Pike MC, McCann SE, Yu H, Xiang YB, et al. Type I and II endometrial cancers: have they different risk factors? J Clin Oncol. 2013;31(20):2607–18. 23733771 10.1200/JCO.2012.48.2596 PMC3699726

[9] Bluher M. Obesity: global epidemiology and pathogenesis. Nat Rev Endocrinol. 2019;15(5):288–98. 30814686 10.1038/s41574-019-0176-8

[10] Wang X, Glubb DM, O’Mara TA. 10 years of GWAS discovery in endometrial cancer: aetiology, function and translation. EBioMedicine. 2022;77:103895. 35219087 10.1016/j.ebiom.2022.103895 PMC8881374

[11] Raglan O, Kalliala I, Markozannes G, Cividini S, Gunter MJ, Nautiyal J, et al. Risk factors for endometrial cancer: an umbrella review of the literature. Int J Cancer. 2019;145(7):1719–30. 30387875 10.1002/ijc.31961

[12] Cheng THT, Thompson DJ, O’Mara TA, Painter JN, Glubb DM, Flach S, et al. Five endometrial cancer risk loci identified through genome-wide association analysis. Nat Genet. 2016;48(6):667–74. 27135401 10.1038/ng.3562 PMC4907351

[13] O’Mara TA, Glubb DM, Amant F, Annibali D, Ashton K, Attia J, et al. Identification of nine new susceptibility loci for endometrial cancer. Nat Commun. 2018;9(1):3166. 30093612 10.1038/s41467-018-05427-7 PMC6085317

[14] Spurdle AB, Thompson DJ, Ahmed S, Ferguson K, Healey CS, O’Mara T, et al. Genome-wide association study identifies a common variant associated with risk of endometrial cancer. Nat Genet. 2011;43(5):451–5. 21499250 10.1038/ng.812 PMC3770523

[15] Painter JN, O’Mara TA, Marquart L, Webb PM, Attia J, Medland SE, et al. Genetic risk score Mendelian randomization shows that obesity measured as body mass index, but not waist: hip ratio, is causal for endometrial cancer. Cancer Epidemiol Biomarkers Prev. 2016;25(11):1503–10. 27550749 10.1158/1055-9965.EPI-16-0147 PMC5093082

[16] Day FR, Thompson DJ, Helgason H, Chasman DI, Finucane H, Sulem P, et al. Genomic analyses identify hundreds of variants associated with age at menarche and support a role for puberty timing in cancer risk. Nat Genet. 2017;49(6):834–41. 28436984 10.1038/ng.3841 PMC5841952

[17] Ruth KS, Day FR, Hussain J, Martinez-Marchal A, Aiken CE, Azad A, Thompson DJ, Knoblochova L, Abe H, Tarry-Adkins JL, et al. Genetic insights into biological mechanisms governing human ovarian ageing. Nature. 2021;596(7872):393–7. 10.1038/s41586-021-03779-7 PMC7611832 34349265

[18] D’Urso S, Arumugam P, Weider T, Hwang LD, Bond TA, Kemp JP, et al. Mendelian randomization analysis of factors related to ovulation and reproductive function and endometrial cancer risk. BMC Med. 2022;20(1):419. 36320039 10.1186/s12916-022-02585-w PMC9623961

[19] Collaborative Group on Epidemiological Studies on Endometrial C. Endometrial cancer and oral contraceptives: an individual participant meta-analysis of 27 276 women with endometrial cancer from 36 epidemiological studies. Lancet Oncol. 2015;16(9):1061–1070. 10.1016/S1470-2045(15)00212-0 26254030

[20] Bycroft C, Freeman C, Petkova D, Band G, Elliott LT, Sharp K, et al. The UK biobank resource with deep phenotyping and genomic data. Nature. 2018;562(7726):203–9. 30305743 10.1038/s41586-018-0579-z PMC6786975

[21] UK Biobank data showcase. https://biobank.ctsu.ox.ac.uk/crystal/browse.cgi.

[22] National Academies of Sciences Engineering and Medicine. Using population descriptors in genetics and genomics research: a new framework for an evolving field. In: Washington (DC): National Academies Press; 2023. 36989389

[23] Kho PF, Mortlock S, Rogers PAW, Nyholt DR, Montgomery GW, Spurdle AB, Glubb DM, O’Mara TA, Consortium ECA, Cons IEG. Genetic analyses of gynecological disease identify genetic relationships between uterine fibroids and endometrial cancer, and a novel endometrial cancer genetic risk region at the WNT4 1p36.12 locus. Hum Genet 2021;140(9):1353–1365. 10.1007/s00439-021-02312-0 34268601

[24] Lloyd-Jones LR, Zeng J, Sidorenko J, Yengo L, Moser G, Kemper KE, et al. Improved polygenic prediction by Bayesian multiple regression on summary statistics. Nat Commun. 2019;10(1):5086. 31704910 10.1038/s41467-019-12653-0 PMC6841727

[25] Zeng J, de Vlaming R, Wu Y, Robinson MR, Lloyd-Jones LR, Yengo L, et al. Signatures of negative selection in the genetic architecture of human complex traits. Nat Genet. 2018;50(5):746–53. 29662166 10.1038/s41588-018-0101-4

[26] Choi SW, Mak TS, O’Reilly PF. Tutorial: a guide to performing polygenic risk score analyses. Nat Protoc. 2020;15(9):2759–72. 32709988 10.1038/s41596-020-0353-1 PMC7612115

[27] Purcell S, Neale B, Todd-Brown K, Thomas L, Ferreira MA, Bender D, et al. PLINK: a tool set for whole-genome association and population-based linkage analyses. Am J Hum Genet. 2007;81(3):559–75. 17701901 10.1086/519795 PMC1950838

[28] Wang X, Kho PF, Ramachandran D, Bafligil C, Amant F, Goode EL, Scott RJ, Tomlinson I, Evans DG, Davidson EJ et al. Multi-trait GWAS identifies a novel endometrial cancer risk locus that associates with testosterone levels. iScience. 2023;26:106590. 10.1016/j.isci.2023.106590 PMC10165198 37168552

[29] Ruth KS, Day FR, Tyrrell J, Thompson DJ, Wood AR, Mahajan A, et al. Using human genetics to understand the disease impacts of testosterone in men and women. Nat Med. 2020;26(2):252–8. 32042192 10.1038/s41591-020-0751-5 PMC7025895

[30] GWAS Catalog. Trait: Age at natural menopause. https://www.ebi.ac.uk/gwas/studies/GCST90320256.

[31] GWAS Catalog. Trait: Sex hormone-binding globulin levels. https://www.ebi.ac.uk/gwas/studies/GCST90012107.

[32] GWAS Catalog. Trait: Total testosterone levels. https://www.ebi.ac.uk/gwas/studies/GCST90012112.

[33] Yang J, Benyamin B, McEvoy BP, Gordon S, Henders AK, Nyholt DR, et al. Common SNPs explain a large proportion of the heritability for human height. Nat Genet. 2010;42(7):565–9. 20562875 10.1038/ng.608 PMC3232052

[34] Yang J, Lee SH, Goddard ME, Visscher PM. GCTA: a tool for genome-wide complex trait analysis. Am J Hum Genet. 2011;88(1):76–82. 21167468 10.1016/j.ajhg.2010.11.011 PMC3014363

[35] Lee SH, Goddard ME, Wray NR, Visscher PM. A better coefficient of determination for genetic profile analysis. Genet Epidemiol. 2012;36(3):214–24. 22714935 10.1002/gepi.21614

[36] Peduzzi P, Concato J, Kemper E, Holford TR, Feinstein AR. A simulation study of the number of events per variable in logistic regression analysis. J Clin Epidemiol. 1996;49(12):1373–9. 8970487 10.1016/s0895-4356(96)00236-3

[37] Rembold CM. Number needed to screen: development of a statistic for disease screening. BMJ. 1998;317(7154):307–12. 9685274 10.1136/bmj.317.7154.307 PMC28622

[38] Kerr KF, Wang Z, Janes H, McClelland RL, Psaty BM, Pepe MS. Net reclassification indices for evaluating risk prediction instruments: a critical review. Epidemiology. 2014;25(1):114–21. 24240655 10.1097/EDE.0000000000000018 PMC3918180

[39] Reay WR, Clarke E, Eslick S, Riveros C, Holliday EG, McEvoy MA, et al. Using genetics to inform interventions related to sodium and potassium in hypertension. Circulation. 2024;149(13):1019–32. 38131187 10.1161/CIRCULATIONAHA.123.065394 PMC10962430

[40] Kundu S, Aulchenko YS, van Duijn CM, Janssens AC. PredictABEL: an R package for the assessment of risk prediction models. Eur J Epidemiol. 2011;26(4):261–4. 21431839 10.1007/s10654-011-9567-4 PMC3088798

[41] Robin X, Turck N, Hainard A, Tiberti N, Lisacek F, Sanchez JC, et al. pROC: an open-source package for R and S+ to analyze and compare ROC curves. BMC Bioinformatics. 2011;12:77. 21414208 10.1186/1471-2105-12-77 PMC3068975

[42] Win AK, Reece JC, Ryan S. Family history and risk of endometrial cancer: a systematic review and meta-analysis. Obstet Gynecol. 2015;125(1):89–98. 25560109 10.1097/AOG.0000000000000563

[43] Johnatty SE, Tan YY, Buchanan DD, Bowman M, Walters RJ, Obermair A, et al. Family history of cancer predicts endometrial cancer risk independently of Lynch Syndrome: implications for genetic counselling. Gynecol Oncol. 2017;147(2):381–7. 28822557 10.1016/j.ygyno.2017.08.011

[44] Davidson EJ, Kitson SJ, McAlpine JN, Mukhopadhyay A, Powell ME, Singh N. Endometrial cancer. Lancet. 2022;399(10333):1412–28. 35397864 10.1016/S0140-6736(22)00323-3

[45] Ryan NAJ, Glaire MA, Blake D, Cabrera-Dandy M, Evans DG, Davidson EJ. The proportion of endometrial cancers associated with Lynch syndrome: a systematic review of the literature and meta-analysis. Genet Med. 2019;21(10):2167–80. 31086306 10.1038/s41436-019-0536-8 PMC8076013

[46] Ryan NAJ, McMahon R, Tobi S, Snowsill T, Esquibel S, Wallace AJ, et al. The proportion of endometrial tumours associated with Lynch syndrome (PETALS): a prospective cross-sectional study. PLoS Med. 2020;17(9):e1003263. 32941469 10.1371/journal.pmed.1003263 PMC7497985

[47] Hingorani AD, Gratton J, Finan C, Schmidt AF, Patel R, Sofat R, et al. Performance of polygenic risk scores in screening, prediction, and risk stratification: secondary analysis of data in the Polygenic Score Catalog. BMJ Med. 2023;2(1):e000554. 37859783 10.1136/bmjmed-2023-000554 PMC10582890

[48] Huntley C, Torr B, Sud A, Rowlands CF, Way R, Snape K, et al. Utility of polygenic risk scores in UK cancer screening: a modelling analysis. Lancet Oncol. 2023;24(6):658–68. 37178708 10.1016/S1470-2045(23)00156-0

[49] Lee A, Mavaddat N, Wilcox AN, Cunningham AP, Carver T, Hartley S, et al. BOADICEA: a comprehensive breast cancer risk prediction model incorporating genetic and nongenetic risk factors. Genet Med. 2019;21(8):1708–18. 30643217 10.1038/s41436-018-0406-9 PMC6687499

[50] Gao C, Polley EC, Hart SN, Huang H, Hu C, Gnanaolivu R, et al. Risk of breast cancer among carriers of pathogenic variants in breast cancer predisposition genes varies by polygenic risk score. J Clin Oncol. 2021;39(23):2564–73. 34101481 10.1200/JCO.20.01992 PMC8330969

[51] Luckett AM, Weedon MN, Hawkes G, Leslie RD, Oram RA, Grant SFA. Utility of genetic risk scores in type 1 diabetes. Diabetologia. 2023;66(9):1589–600. 37439792 10.1007/s00125-023-05955-y PMC10390619

[52] Luo J, Chlebowski RT, Hendryx M, Rohan T, Wactawski-Wende J, Thomson CA, et al. Intentional weight loss and endometrial cancer risk. J Clin Oncol. 2017;35(11):1189–93. 28165909 10.1200/JCO.2016.70.5822 PMC5455602

[53] Zhang X, Rhoades J, Caan BJ, Cohn DE, Salani R, Noria S, et al. Intentional weight loss, weight cycling, and endometrial cancer risk: a systematic review and meta-analysis. Int J Gynecol Cancer. 2019;29(9):1361–71. 31451560 10.1136/ijgc-2019-000728 PMC6832748

[54] Rask-Andersen M, Ivansson E, Hoglund J, Ek WE, Karlsson T, Johansson A. Adiposity and sex-specific cancer risk. Cancer Cell. 2023;41(6):1186-1197 e1184. 37311415 10.1016/j.ccell.2023.05.010

[55] Dominguez-Valentin M, Sampson JR, Seppala TT, Ten Broeke SW, Plazzer JP, Nakken S, et al. Cancer risks by gene, age, and gender in 6350 carriers of pathogenic mismatch repair variants: findings from the Prospective Lynch Syndrome Database. Genet Med. 2020;22(1):15–25. 31337882 10.1038/s41436-019-0596-9 PMC7371626

[56] Parkin CJ, Bell SW, Mirbagheri N. Colorectal cancer screening in Australia: an update. Aust J Gen Pract. 2018;47(12):859–63. 31212405 10.31128/AJGP-01-18-4472

[57] Sollis E, Mosaku A, Abid A, Buniello A, Cerezo M, Gil L, et al. The NHGRI-EBI GWAS Catalog: knowledgebase and deposition resource. Nucleic Acids Res. 2023;51(D1):D977–85. 36350656 10.1093/nar/gkac1010 PMC9825413

[58] GWAS Catalog. Trait: Endometrial Cancer. https://www.ebi.ac.uk/gwas/studies/GCST006464.

