# Supplementary material for: Risk stratification for endometrial cancer: independent and joint effects of polygenic risk score and body mass index in 129,829 UK Biobank participants
Source: BMC Med. 2026 Feb 10;24:26. doi: 10.1186/s12916-025-04570-5 (PMC12888353; doi:10.1186/s12916-025-04570-5)
Supplement: Supplementary file 1 — Additional file 1: Table S1. Baseline characteristics of the 129,829 UK Biobank female participants included in the study, stratified by endometrial cancer case status. Characteristics include demographic data, anthropometric measurements, reproductive factors, and hormone levelsand their corresponding polygenic scores. [file 12916_2025_4570_MOESM1_ESM.pdf]

Table S1. Baseline characteristics of participants in the UK Biobank for the final analysis cohort.

Baseline characteristics	Overall	Cases	Controls	P-value
Number of female participants	129829	956 (0.7%)	128873 (99.3%)	
Age at initial assessment, years (SD)	55.7 (8.0)	59.5 (6.6)	55.7 (8.0)	$< 2.2 \times 10^{-16}$
BMI (SD), continuous	26.9 (5.1)	30.5 (6.9)	26.9 (5.1)	$< 2.2 \times 10^{-16}$
BMI categories[a]				
BMI < 25kg/m2	53362 (41.1%)	206 (21.5%)	53156 (41.2%)	$< 2.2 \times 10^{-16}$
25kg/m2 <= BMI < 30kg/m2	47234 (36.4%)	332 (34.7%)	46902 (36.4%)	
BMI >= 30kg/m2	29233 (22.5%)	418 (43.7%)	28815 (22.4%)	
Age at menarche, years (SD)	13.0 (1.6)	12.7 (1.6)	13.0 (1.6)	1.8×10^{-9}
Age at menopause, years (SD)	50.4 (4.4)	51.7 (4.3)	50.4 (4.4)	$< 2.2 \times 10^{-16}$
Number of post-menopausal females (%)	70564 (54.7%)	737 (77.1%)	69827 (54.1%)	
Number of pre-menopausal females (%)	55277 (42.6%)	181 (18.9%)	55096 (42.8%)	
Missing (%)	3988 (3.1%)	38 (4.0%)	3950 (3.1%)	
Age at menopause PGS[b] (SD)	0 (1.43)	0.17 (1.44)	0 (1.43)	2.5×10^{-4}
SHBG levels (nmol/L; SD)	62.5 (30.7)	50.7 (26.1)	62.6 (30.7)	$< 2.2 \times 10^{-16}$
Number of females that had detectable SHBG levels	111217 (85.7%)	828 (86.6%)	110389 (85.7%)	
Missing (%)	18612 (14.3%)	128 (13.4%)	18484 (14.3%)	
SHBG PGS[b] (SD)	0 (0.22)	-0.05 (0.23)	0 (0.22)	1.1×10^{-10}
Testosterone levels (nmol/L; SD)	1.1 (0.6)	1.2 (0.6)	1.1 (0.6)	7.6×10^{-7}
Number of females that had detectable testosterone levels	104550 (80.5%)	799 (83.6%)	103751 (80.5%)	
Missing (%)	25279 (19.5%)	157 (16.4%)	25122 (19.5%)	
Testosterone PGS[b] (SD)	0 (0.34)	0.05 (0.36)	0 (0.34)	3.8×10^{-6}
Number of live births (SD), count	1.8 (1.2)	1.7 (1.2)	1.8 (1.2)	0.1
Number of live births categories[a]				
0 (%)	24214 (18.7%)	217 (22.7%)	23997 (18.6%)	4.6×10^{-3}
1 (%)	17175 (13.2%)	114 (11.9%)	17061 (13.2%)	

> 1 (%)	88440 (68.1%)	625 (65.4%)	87815 (68.1%)	
Ever taken oral contraceptive pill[a]				
Yes (%)	109047 (84.0%)	695 (72.7%)	108352 (84.1%)	$< 2.2 \times 10^{-16}$
No (%)	20782 (16.0%)	261 (27.3%)	20521 (15.9%)	

Abbreviations - SD: standard deviation; BMI: body mass index; PGS: polygenic score; SHBG: sex hormone binding globulin

[a]Percentages may not add up to 100% due to rounding;

[b]PGS (polygenic scores) of age at menopause, SHBG, and testosterone were shifted to mean zero
