# Supplementary material for: Risk stratification for endometrial cancer: independent and joint effects of polygenic risk score and body mass index in 129,829 UK Biobank participants
Source: BMC Med. 2026 Feb 10;24:26. doi: 10.1186/s12916-025-04570-5 (PMC12888353; doi:10.1186/s12916-025-04570-5)
Supplement: Supplementary file 2 — Additional file 2: Supplementary Figures. Fig S1–Distribution of the endometrial cancer polygenic risk scoreby unrelated female cases and controls of European genetic ancestry in UK Biobank. Fig S2–Joint association of genetic risk and BMI with endometrial cancer risk with additional adjustment for continuous BMI. [file 12916_2025_4570_MOESM2_ESM.pdf]

Additional File 2: Supplementary Figures

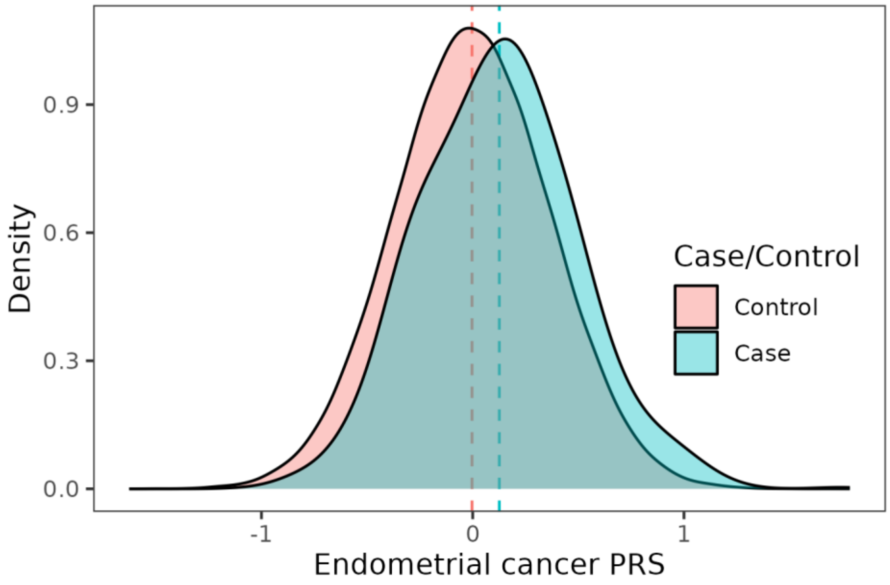

Figure S1. The distribution of endometrial cancer PRS by unrelated female cases and controls of European genetic ancestry in the UK Biobank.

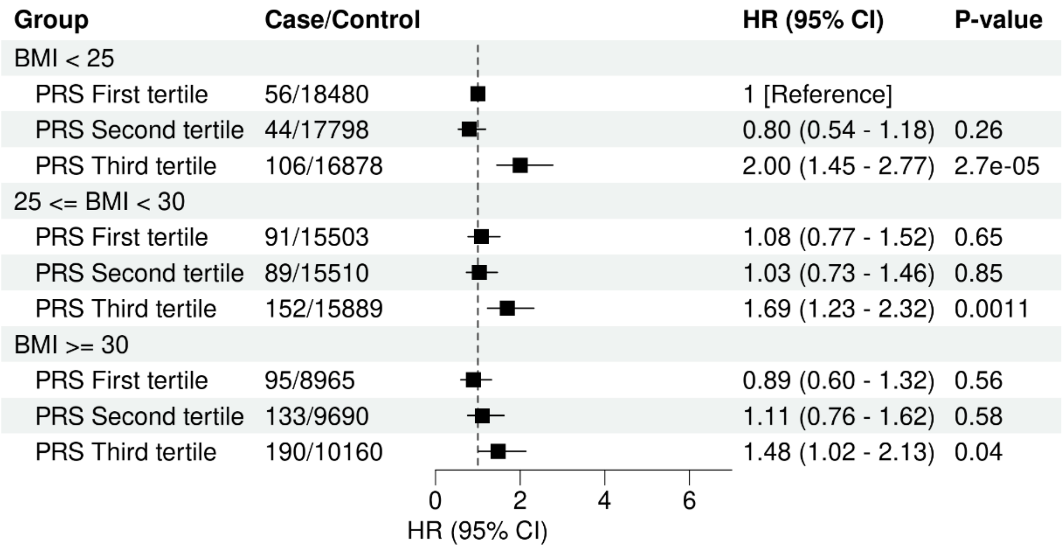

Figure S2. The joint association of genetic risk and BMI with endometrial cancer with additional adjustment for continuous BMI
