# Supplementary material for: Risk stratification for endometrial cancer: independent and joint effects of polygenic risk score and body mass index in 129,829 UK Biobank participants
Source: BMC Med. 2026 Feb 10;24:26. doi: 10.1186/s12916-025-04570-5 (PMC12888353; doi:10.1186/s12916-025-04570-5)
Supplement: Supplementary file 3 — Additional file 3: Supplementary Note. Sensitivity analyses using reported age of menopause and measured SHBG and testosterone levels instead of polygenic scores for these variables. Includes Table 1 comparing discriminative performance of prediction models and Figs. 1–2 demonstrating that BMI and PRS independently associate with endometrial cancer risk without evidence of interaction. [file 12916_2025_4570_MOESM3_ESM.pdf]

Supplementary Note

Results from sensitivity analyses provided below using reported age of menopause and measured SHBG and testosterone levels instead of PGS for these variables. Total numbers included in analysis with all variables available: 557 incident endometrial cancer cases and 50,303 controls. Similar AUC values were observed in this smaller dataset, and for measured risk factors (Table 1). The difference between epidemiological and integrative models remained statistically significant (Z = -3.9466, p-value = 7.929e-05). The continuous net reclassification improvement for endometrial cancer prediction was 0.29 (95% CI 0.21-0.37; $P < 1 \times 10^{-4}$), similar to results using the PGS for risk factors (NRI = 0.25).

Table 1. Discriminative performance of endometrial cancer risk factors and prediction models in UK Biobank. Epidemiological model incorporated established risk factors (BMI, age at menarche, parity, oral contraceptive use, menopause age, SHBG, and testosterone); integrative model combining PRS with the epidemiological model. All models were adjusted for age at initial visit, assessment centre and the first ten principal components.

Prediction Model	AUC	95% CI	Sensitivity (%)	Specificity (%)	PPV (%)	NPV (%)
Endometrial cancer PRS	0.650	0.628-0.672	60.7	60.6	1.7	99.4
BMI (continuous)	0.709	0.687-0.731	65.3	64.9	2.0	99.5
Number of live births	0.609	0.586-0.631	61.4	54.2	1.5	99.3
Age of menarche	0.618	0.596-0.640	62.3	54.8	1.5	99.3
Age of menopause	0.633	0.611-0.657	62.7	58.9	1.6	99.4
OCP use (never/ever)	0.619	0.597-0.642	59.6	57.8	1.5	99.3
SHBG level	0.669	0.648-0.690	66.2	59.5	1.7	99.4
Testosterone level	0.619	0.597-0.641	61.2	56.0	1.5	99.3
Epidemiological model	0.736	0.715-0.757	67.1	67.6	2.2	99.5
Integrative model	0.753	0.733-0.773	66.2	70.5	2.4	99.5

Abbreviations – AUC: area under the receiver operator curve; CI: confidence interval; PPV: positive predictive value; NPV: negative predictive value; PRS: polygenic risk score; BMI: body mass index; OCP: oral contraceptive pill; SHBG: sex hormone binding globulin.

There was no evidence to support violation of the proportional hazard assumption in this sensitivity analysis (global test $P = 0.21$). BMI and PRS remained independently associated with endometrial cancer risk in sensitivity analysis (**Figure 1**). There was no evidence of interaction between BMI and endometrial cancer PRS (P for interaction = 0.42). When endometrial cancer PRS and BMI were treated as continuous variables, an increase of one standard deviation of endometrial cancer PRS was associated with 2.58 -fold risk of endometrial cancer (95% CI, 2.05-3.25; $P = 4.44 \times 10^{-16}$), while a one standard deviation increase of BMI (5.0 kg/m^2) was associated with a 1.59-fold risk (95% CI, 1.48-1.70; $P = 3.04 \times 10^{-41}$). Results observed in joint association analyses using measure risk factors in this reduced cohort were consistent with the original findings, with greatest risk for endometrial cancer among the top PRS tertile in each BMI group.

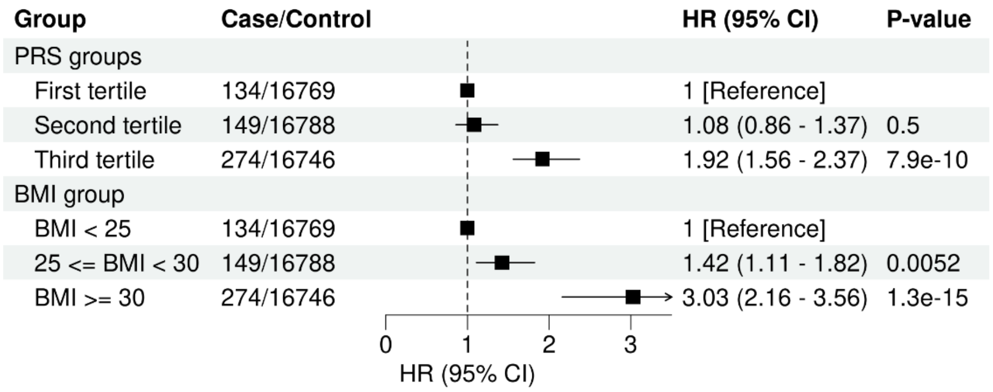

Figure 1. Multivariable-adjusted effects of endometrial cancer polygenic risk score (PRS) and BMI on endometrial cancer risk. Multivariable models were adjusted for either PRS or BMI group and additionally adjusted for age at menarche, age at menopause, number of live births, ever taken oral contraceptive pill, measured levels of SHBG and testosterone, as well as age at initial assessment and the top 10 genetic principal components.

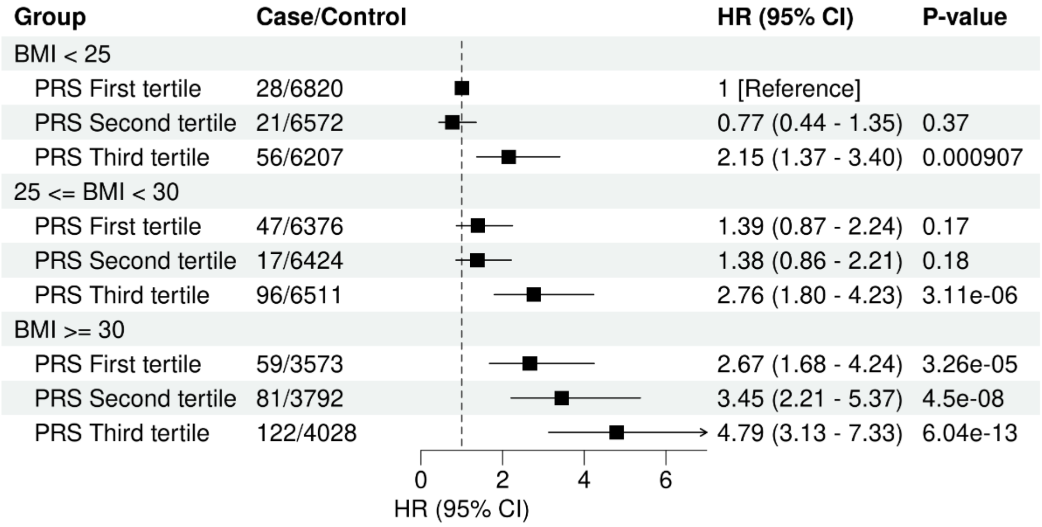

Figure 2. The joint association of genetic risk and BMI with endometrial cancer. Multivariable models were adjusted for age at initial assessment, age at menarche, age at menopause, number of live births, ever taken oral contraceptive pill, measured levels of SHBG and testosterone and the top 10 genetic principal components. The dashed vertical line indicates a Hazard ratio of 1.
