# Supplementary material for: Risk stratification for endometrial cancer: independent and joint effects of polygenic risk score and body mass index in 129,829 UK Biobank participants
Source: BMC Med. 2026 Feb 10;24:26. doi: 10.1186/s12916-025-04570-5 (PMC12888353; doi:10.1186/s12916-025-04570-5)
Supplement: Supplementary file 4 — Additional file 4: Supplementary Note. Strengthening the Reporting of Observational Studies in Epidemiology checklist. [file 12916_2025_4570_MOESM4_ESM.pdf]

STROBE Checklist (Completed)

Item No	Recommendation	How Addressed in Manuscript
1a	Indicate the study's design with a commonly used term in the title or abstract	Described as a cohort study in the abstract (Abstract page 1-2)
1b	Provide in the abstract an informative and balanced summary of what was done and what was found	Abstract includes background, methods, results, conclusions (pages 1-2)
2	Explain the scientific background and rationale	Background on obesity, PRS, and rationale for risk stratification (Background pages 3-4)
3	State specific objectives, including hypotheses	Objectives: assess independent and joint effects of BMI and PRS. Evaluate prediction model (Abstract & Background pages 3-4)
4	Present key elements of study design early	Prospective cohort study (Methods page 4)
5	Describe setting, locations, dates	UK Biobank; recruitment 2006–2010; follow-up to 2021 (Methods pages 4-10)
6a	Eligibility criteria, sources, selection methods; methods of follow-up	Inclusion/exclusion criteria, case ascertainment, controls and exclusions (pages 4-5; Fig. 1)
6b	Matching criteria (if applicable)	Not applicable (no matching used)
7	Define all outcomes, exposures, predictors, confounders	Detailed in Methods (pages 4-10; Table 1)
8	Data sources/measurement	Phenotypes, genotypes, PRS derivation (Methods pages 4-10)
9	Efforts to address bias	Excluded prevalent cases, adjusted covariates, proportional hazards assumption tested (Methods pages 4-10)
10	Study size	Events-per-variable calculation described (page 9)
11	Handling of quantitative variables	BMI continuous & categorical, PRS tertiles/percentiles (Methods pages 4-10)
12a	Statistical methods, confounding control	Regression, Cox, AUC, NRI, bootstrap (Methods pages 8-10)
12b	Methods for subgroups/interactions	BMI × PRS tested (page 10)
12c	Missing data handling	Excluded participants with missing covariates; proportions reported (Table 1; pages 4-5)
12d	Loss to follow-up	Not applicable (registry-linked outcomes)
12e	Sensitivity analyses	Continuous BMI/PRS, additional adjustment (Methods page 9, Supplementary Fig. 2, Additional File 3)
13a	Report numbers at each stage	Numbers at each stage (Methods pages 5-6; Fig. 1)

13b	Reasons for non-participation	Exclusions described (withdrawals, missing data, prevalent cases) (Methods pages 5-6)
13c	Flow diagram	Fig. 1 provided
14a	Descriptive data of participants	Table 1 and Supplementary Table 1, Results (pages 10-11)
14b	Numbers with missing data	Reported in Table 1 footnotes
14c	Follow-up time	Summarised in Methods (pages 4-10)
15	Outcome data	956 incident endometrial cancer cases reported (Results page 10)
16a	Unadjusted and adjusted estimates, precision	HRs, ORs, AUCs, adjusted estimates with CIs (Tables 2-3; Figs. 2-3; Results pages 10-13)
16b	Report category boundaries	BMI (<25, 25–30, ≥30); PRS tertiles/percentiles (Methods pages 9)
16c	Translate relative risk into absolute risk if relevant	Absolute risk and NNS provided (Table 2, page 11)
17	Report subgroup/interaction/sensitivity analyses	Stratified, sensitivity analyses described (Results pages 12-13; Supplementary Fig. 2, Additional File 3)
18	Summarise key results	Results summarised relative to aims (pages 14)
19	Limitations	External validation, ancestry, BMI single timepoint, modest AUC (pages 16-17)
20	Interpretation	Balanced discussion, results discussed in context of prior evidence, limitations clear (pages 14-18)
21	Generalisability	Discussed limited to European ancestry, need diverse validation (page 16-17)
22	Funding sources and role	Fully reported (Declarations page 20)
